# Assessing reproducibility in association studies

**DOI:** 10.7554/eLife.46757

**Published:** 2019-04-25

**Authors:** Hugo Schnack

**Affiliations:** Department of PsychiatryUMC UtrechtUtrechtThe Netherlands

**Keywords:** brain connections, structural brain-behavior associations, inter-individual differences, behaviors, psychological traits, reproducibility, Human

## Abstract

Research that links brain structure with behavior needs more data, better analyses, and more intelligent approaches.

**Related research article** Kharabian Masouleh S, Eickhoff SB, Hoffstaedter F, Genon S, Alzheimer’s Disease Neuroimaging Initiative. 2019. Empirical examination of the replicability of associations between brain structure and psychological variables. *eLife* **8**:e43464. doi: 10.7554/eLife.43464

Scientists have always been eager to understand how complex thoughts and behaviors emerge from the intricate networks of neurons found in our brains. For instance, there appears to be a (weak) association between intelligence and total brain volume (Pietschnig et al., 2015), but also between intelligence and the dendritic size of pyramidal neurons (Goriounova et al., 2018). Yet, these relations do not provide a true insight into how individual differences in intelligence or in other behaviors emerge. Just as examining one component in a car, or weighing the whole car, will tell us relatively little about the overall performance of the vehicle, simply looking at individual neurons, or calculating the volume of a brain, will not tell the whole story about a person.

This is because brain areas and structures interact with each other and work in synergy to create and influence behavior. New techniques such as magnetic resonance imaging (MRI) have made it possible to start exploring the way a specific behavior trait is linked to the brain. In particular, many new associations between behavior and brain structure have been revealed with mass-univariate approaches, which divide the brain into small 3D units called voxels, and then map the relationship between behavior and each of these voxels using univariate statistical tests such as ANOVAs or t-tests (Ashburner and Friston, 2000; Kanai and Rees, 2011). However, it has been difficult to replicate some of the findings obtained through these methods (Figure 1).

**Figure 1.**
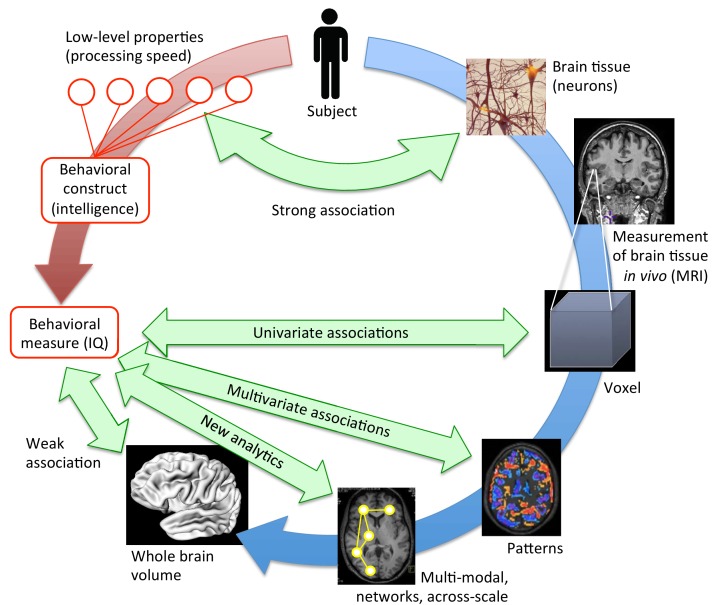
Bringing together brain structure and behavioral traits. Several ways exist to examine how the structural properties of the brain (in blue) underpin complex behavioral traits such as intelligence (in red). At a low, neuronal level, some connections can be drawn between the characteristics of neurons and processing speed. To study more complex behavioral constructs, the brain can be examined at different levels. MRI scans provide averaged information about neural tissue throughout the brain at the scale of the millimeter (voxel; right, middle). This information can then be correlated with high-level behavioral measures using mass-univariate associations. While these structural brain -behavior associations are stronger than those obtained when looking at the whole brain volume (lower left), many have not been replicated (Kharabian Masouleh et al., 2019). However, multivariate analyses (lower right), and innovative, multi-modal analyses that work at the scale of networks (bottom), are expected to provide the strongest associations. They may hold the key to understanding how brain structure underpins psychological measures like intelligence.

Now, in eLife, Shahrzad Kharabian Masouleh, Simon Eickhoff, Felix Hoffstaedter and Sarah Genon from Research Centre Jülich and Heinrich Heine University Düsseldorf, along with the Alzheimer’s Disease Neuroimaging Initiative (ADNI), report new insights into these problems (Kharabian Masouleh et al., 2019). The team used common mass-univariate methods on two relatively large samples of 371 and 466 individuals to examine structural brain-behavior (SBB) associations with 36 psychological measures. These analyses revealed that it is difficult to pinpoint relationships between brain structures and behavioral traits, and that these relationships often occupy different locations between samples, making them hard to replicate.

What could explain and even fix this lack of reliability in SBB association studies? One issue is that psychological variables such as happiness or intelligence are complex constructs that rely on many different neuronal processes. Such diffuse measures may therefore yield unsteady correlations. In contrast, Kharabian Masouleh et al. showed that age yielded widespread and highly reproducible associations with brain structure. This may be because this ‘hard’, uncomplicated measure affects the organ consistently across different scales, from neurons to brain areas.

Another problem is that the statistical methods used in mass-univariate analyses cannot model the synergy between different parts of the brain, or that the brain may organize behaviors differently between individuals. An illustration of this limitation is that the current work failed to replicate association peaks, whereby small regions of brain tissue show important SBB associations (also discussed by Kanai, 2016). Instead, multivariate pattern recognition techniques can detect associations between behavior and structural patterns in the brain, making possible to identify groups of voxels that change together with variations in intelligence.

Nowadays, machine learning studies use these multivariate analyses. These new approaches can also independently select relevant features and take into account both interactions between brain structures and heterogeneity amongst individuals. In addition, it has become standard procedure to try to replicate results within the discovery set (by cross-validation) and in independent test samples (for example, Dwyer et al., 2018).

Using more data will also protect against irreproducibility and improve generalizability. Small samples are prone to chance findings, but large datasets help to reduce noise and sampling variance while also capturing more heterogeneity (Schnack and Kahn, 2016). Finally, recommendations point towards making as much information as possible public, for instance by publishing null findings and sharing raw data (as done by, for example, the UK Biobank). If this is not possible, the results of the analyses – the statistical brain maps – should be released so they can be used in meta-analyses, for example.

Voxel-based measures work at the scale of the millimeter and therefore ignore the details of the many neurons present in the voxels. On the other hand, it is now possible to zoom in on cortical layers using 3T MRI (Ferguson et al., 2018). This could be a first step towards examining the living brain with a resolution normally only accessible through post mortem research.

Further improvements could come from going beyond measuring volumes, for instance by starting to assess connectivity at different scales (Scholtens and van den Heuvel, 2018), by employing spectroscopic measures, or by combining the two. Yet, more rigorous innovations may still be necessary: to finally understand rich and multifaceted concepts, such as the emergence of intelligence, scientists will need to design equally complex approaches to analyze the brain in a more clever way.
